# Trustworthy or flawed clinical prediction rule?

**DOI:** 10.1186/s13054-018-1961-9

**Published:** 2018-02-08

**Authors:** Anders Granholm, Anders Perner, Aksel Karl Georg Jensen, Morten Hylander Møller

**Affiliations:** 1 Department of Intensive Care 4131, Copenhagen University Hospital—Rigshospitalet, Blegdamsvej 9, 2100 Copenhagen, Denmark; 2 Centre for Research in Intensive Care, Blegdamsvej 9, 2100 Copenhagen, Denmark; 3 Section of Biostatistics, University of Copenhagen, Øster Farimagsgade 5, 1014 Copenhagen, Denmark

We read with interest the recently published paper by Hilder et al. [1], where the authors present the PRESET-Score, a new clinical prediction rule for patients with acute respiratory distress syndrome treated with extracorporeal membrane oxygenation (ECMO). While the topic is clinically relevant and interesting, we are worried that spurious findings, biased results, and overstated findings are presented.

First, both the new and the four existing scores assessed are at high risk of being underpowered. Multivariable risk prediction models should be based on an effective sample size (lowest number of events/non-events) of at least 10, often more, per predictor variable assessed [2, 3]. Using 11 variables and 41 non-events (3.7 per predictor) results in overfitting of the development sample and inflated performance estimates [2]. This will be evident upon use of the score in other populations.

Second, comparing the performance of the new score with four existing scores using the development dataset is against recommendations [2], as this is biased to favor the new score due to overfitting. For comparison with other scores, an independent cohort not used to develop *any* of the scores must be used [2].

Third, internal validation is performed to quantify overfitting, and should be done by bootstrap resampling of the development dataset [2]. The authors state that they used logistic regression analysis to “reassess” the score, which essentially is a *recalibration* resulting in a new model generating new predictions. This is neither internal nor external validation, which requires assessment of predictions made by the score *without* modifications in a new sample [2].

Fourth, it is recommended to assess calibration by graphical methods or regressions of the predicted versus observed outcomes [2, 4], not by the Hosmer-Lemeshow *Ĉ*-test, as *P* > 0.05 is more likely to indicate lack of power than proper model fit when used on small samples.

While we agree that clinical prediction rules may be valuable for clinicians considering ECMO, it is a prerequisite that such scores are developed and validated using appropriate methodology [2] and sufficient sample sizes, and that all relevant features are transparently reported with adequate discussion of the limitations [5]. Developing and sufficiently validating a clinical prediction rule for this highly selected patient group likely requires a large, multicentre collaboration to ensure trustworthy predictions that will benefit patients and relatives, the healthcare system, researchers, and society.

## Abbreviations

ECMO: Extracorporeal membrane oxygenation
